# Endometrial stem/progenitor cells and their roles in immunity, clinical application, and endometriosis

**DOI:** 10.1186/s13287-021-02526-z

**Published:** 2021-08-23

**Authors:** Yue Kong, Yang Shao, Chunxia Ren, Gong Yang

**Affiliations:** 1 Cancer Institute, Fudan University Shanghai Cancer Center, Shanghai, 200032 China; 2 Department of Oncology, Shanghai Medical College, Fudan University, Shanghai, 200032 China; 3 Center for Reproductive Medicine, Shuguang Hospital Affiliated to Shanghai University of Traditional Chinese Medicine, Shanghai, 200120 China; 4 Central Laboratory, The Fifth People’s Hospital of Shanghai Fudan University, Shanghai, 200240 China

**Keywords:** Stem cells, Endometriosis, Human endometrium, Immunology

## Abstract

Endometrial stem/progenitor cells have been proved to exist in periodically regenerated female endometrium and can be divided into three categories: endometrial epithelial stem/progenitor cells, CD140b^+^CD146^+^ or SUSD2^+^ endometrial mesenchymal stem cells (eMSCs), and side population cells (SPs). Endometrial stem/progenitor cells in the menstruation blood are defined as menstrual stem cells (MenSCs). Due to their abundant sources, excellent proliferation, and autotransplantation capabilities, MenSCs are ideal candidates for cell-based therapy in regenerative medicine, inflammation, and immune-related diseases. Endometrial stem/progenitor cells also participate in the occurrence and development of endometriosis by entering the pelvic cavity from retrograde menstruation and becoming overreactive under certain conditions to form new glands and stroma through clonal expansion. Additionally, the limited bone marrow mesenchymal stem cells (BMDSCs) in blood circulation can be recruited and infiltrated into the lesion sites, leading to the establishment of deep invasive endometriosis. On the other hand, cell derived from endometriosis may also enter the blood circulation to form circulating endometrial cells (CECs) with stem cell-like properties, and to migrate and implant into distant tissues. In this manuscript, by reviewing the available literature, we outlined the characteristics of endometrial stem/progenitor cells and summarized their roles in immunoregulation, regenerative medicine, and endometriosis, through which to provide some novel therapeutic strategies for reproductive and cancerous diseases.

## Introduction

Endometrium can be divided into shallow and deep layers based on the structure. The shallow layer is called functional layer that experiences periodic changes of proliferation, secretion, and shedding under the regulation of hormones. The deep layer is named as basal layer. The basal layer owns strong proliferation and repair abilities without falling off during the menstrual period but generates new functional layers. The periodic endometrial regeneration implies the presence of stem/progenitor cells in the endometrium. Gargett et al. first revealed the existence of adult stem/progenitor cells in endometrium by identification of rare clonogenic cells or colony-forming units (CFUs) from purified single-cell suspensions of hysterectomy tissues in 2004 [1]. Since then, the study of endometrial stem/progenitor cells has been highly developed. At present, based on cell types and identification techniques, endometrial stem/progenitor cell population is defined as CD140b^+^CD146^+^ or SUSD2^+^ endometrium-derived mesenchymal stem cells (eMSCs), endometrial epithelial stem/progenitor cells, and side population cells (SPs) [2–4], whereas those derived from menstrual blood are called menstrual stem cells (MenSCs).

Endometriosis is defined as the growth and infiltration of endometrial tissue (glands and stroma) outside the uterine cavity with the typical symptom of periodic bleeding, which causes infertility, pain, nodules, and masses [5]. A most widely accepted hypothesis for the pathogenesis of endometriosis first proposed by Sampson et al. in 1927 is that the endometrial glandular epithelium and stromal cells flow within the menstrual blood and enter the pelvic cavity through the fallopian tubes. These cells may invade, grow and spread in the ovary and the adjacent pelvic peritoneum tissues, to eventually form the pelvic endometriosis [6]. This theory is called retrograde menstruation (RM), but it still cannot explain why only 6–10% of the reproductive age women with RM develop into endometriosis [7]. The concept of stem cells may well explain the low incidence of endometriosis in patients with RM because the abnormal endometrial stem/progenitor cells from just a few patients enter the pelvic cavity to cause endometriotic lesions [2, 8–11].

In this review, we collected the recent advances in the identification and characterization of adult stem/progenitor cells in female endometrium and  summarized the cell-based therapy and immunoregulation of endometrial stem/progenitor cells. We also outlined the signaling pathways and molecular mechanisms involved in endometrial stem/progenitor cell populations. The physiological/pathological roles of bone marrow-derived and endogenous stem/progenitor cells in endometriosis are also analyzed. Finally, we proposed that MenSCs are the most promising candidates for the stem cell-based therapy. The investigation of the molecular mechanisms of stem/progenitor cells in the development of endometriosis may provide some novel strategies for molecular therapy of reproductive and cancerous diseases.

## Multiple populations of stem/progenitor cells in endometrium

### CD140b^+^CD146^+^ eMSCs

**Fig. 1:**
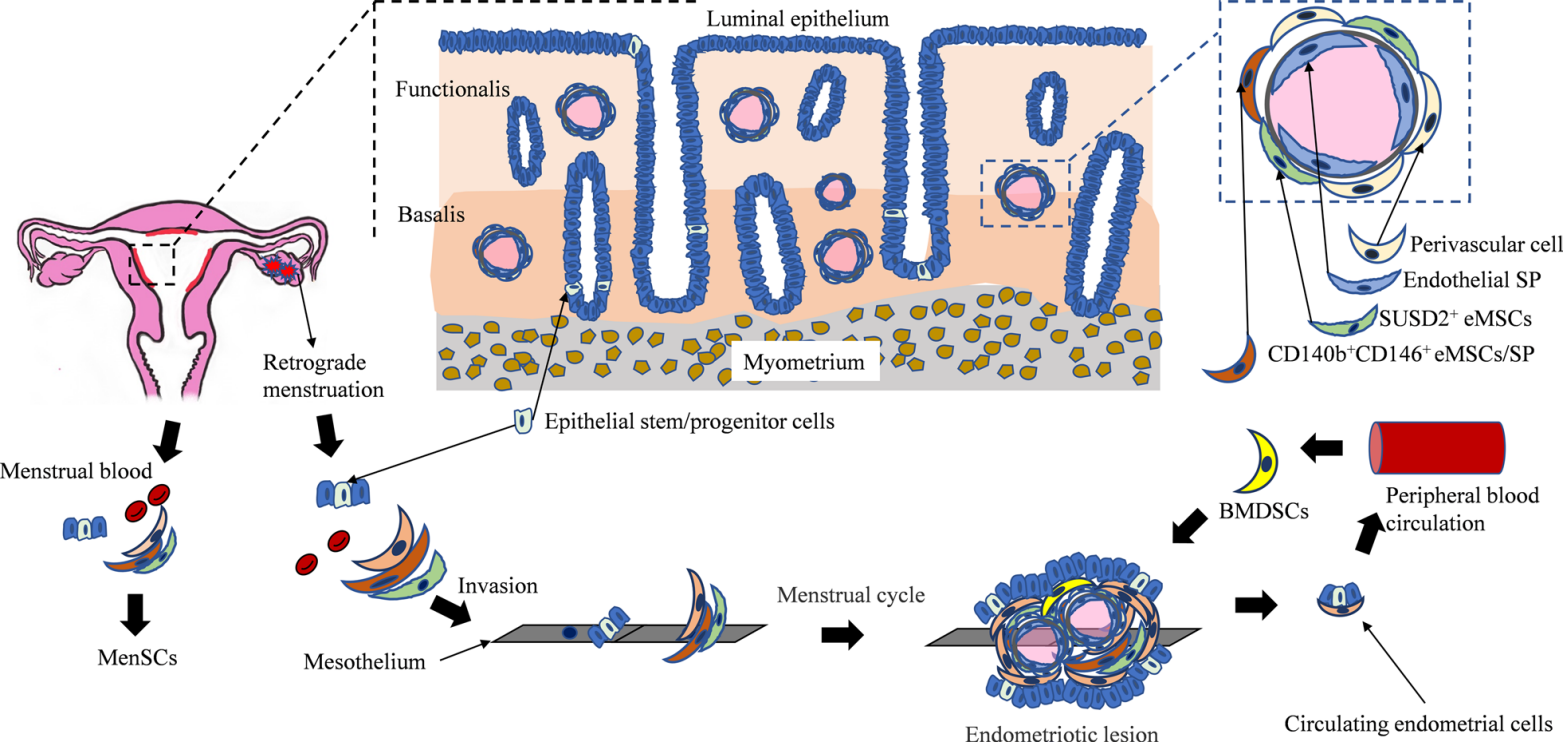
Schematic diagram illustrates the localization of endometrial stem/progenitor cells and the hypothesis that stem cells in RM, BMDSCs and CECs may be involved in the development of endometriosis. CD140b^+^CD146^+^ eMSCs are located perivascularly in both the functionalis and basalis. SUSD2^+^ eMSCs are also perivascular cells. Epithelial progenitor cells are a subset of SSEA-1^+^ cells located at the bottom of basalis, and may form individual colonies. Endometrial SPs are composed of heterogeneous populations, including endothelial cells and CD140b^+^CD146^+^ eMSCs. Endometrial stem/progenitor cells in RM may be the cellular source of primary endometriotic lesions. Abnormal endometrial stem/progenitor cells in RM enter the pelvic cavity and invade the mesothelium. On one hand, endometriotic cells secrete cytokines (such as CXCL12) to attract limited BMDSCs in blood circulation and implant them in the ectopic lesions. On the other hand, endometriotic cells enter the blood circulation to cause distant infiltration

The CD146^+^CD140b^+^ population is located at the perivascular region in both functional and basal layers and can differentiate into osteogenic, myogenic, adipogenic, and chondrogenic lineages, as well as fibroblasts and smooth muscle cells [12–14] (Fig. 1). Mesenchymal stem cell (MSC) markers CD29, CD44, CD73, CD90, CD105, but not endothelial or hemopoietic markers CD31, CD34, and CD45, are expressed in this population [15] (Table 1). The percentage and clonal capacity of CD140b^+^CD146^+^ cells are constant at different stages of the menstrual cycle (menstrual, proliferative, and secretory phases). However, compared with the secretory stage, CD140b^+^CD146^+^ cells from the menstrual endometrium experience more rounds of the self-renewal, suggesting that CD140b^+^CD146^+^ cells may be activated during menstruation to promote the periodic regeneration of the endometrium. More CD140b^+^CD146^+^ cells can be detected in the deeper portion of the endometrium than in the superficial layer, but their clonogenic and self-renewal activities remain similarly [16]. Gene expression profiling revealed that 1518 and 762 genes are differentially and significantly expressed between CD140b^+^CD146^+^ cells and endothelial cells, or between CD140b^+^CD146^+^ cells and stromal fibroblasts, respectively [13]. In addition, CD140b^+^CD146^+^ cells highly express genes involved in angiogenesis, steroid hormone/hypoxia responses, immunomodulation, inflammation, cell communication, and proteolysis/inhibition, and display the increased expression of Notch, IGF, TGF-β, Hedgehog, and G protein-coupled receptor signaling molecules compared with CD140b^+^CD146^−^ cells [13]. Co-culture of endometrial cells (epithelial or stromal) derived from menstruation with CD140b^+^CD146^+^ eMSCs enhances the cloning and self-renewal activities of CD140b^+^CD146^+^ eMSCs. Co-culture of CD140b^+^CD146^+^ cells with the endometrial niche cell conditioned media containing the high levels of interleukin 6, C-X-C motif ligand 1 (CXCL1) and CXCL5 may increase the proliferation and self-renewal abilities of CD140b^+^CD146^+^ eMSCs [17].

**Table 1:** Surface marker phenotype and in vitro/in vivo differentiation of human endometrial stem cells

Cell type	Positive marker	Negative marker	In vitro and in vivo differentiation	References
MenSCs	CD73, CD90, CD105, CD13, CD44, CD29, CD9, CD44, CD41a, CD59	CD19, CD34, CD45, CD117, CD130, HLA-DR	Adipocytes, osteocytes, cardiomyocytes, neurocytes, respiratory epithelial cells, endothelial cells, myocytes, hepatic cells, pancreatic cells, and germ-like cell	[42, 43]
Endometrial SPs of epithelial origin	CD9, CD90, CD105, CD73, CD45, CD34, CD31, CD133, stro-1	CD9, CD13	Adipocytes, osteocytes	[96, 97]
Endometrial SPs from the stromal compartment	Vimentin, CD90, CD73, CD45, CD34, CD31, CD133, stro-1	CD9, CD13, CD105, ERα, PR	Adipocytes, osteocytes	[97]
SUSD2^+^ eMSCs	CD29, CD44, CD73, CD90, CD105, CD117, CD140b, CD146, and STRO-1, NTPDase2	CD31, CD45	Adipocytes, osteocytes, chondrocytes, myocytes, endothelial cells	[12]
CD140b^+^CD146^+^ eMSCs	CD29, CD44, CD73, CD90, CD105, CD140b, CD146	CD31, CD34, CD45	Osteocytes, myocytes, adipocytes, chondrocytes, fibroblasts and smooth muscle cell	[12, 14, 15]
CD146^+^ cells	CD10, CD13, CD44, CD73, CD90, and CD105	CD31, CD34, CD45, CD56, CD144, CD9	Adipocytes, osteoblasts, and neuron-like cells, glial-like cells	[19, 21]
Epithelial stem/progenitor cells	N-cadherin, SSEA-1, Axin 2		Entire complement of glandular lineages, endometrial organoids	[104, 107, 150]

*MenSCs* menstrual stem cells, *SPs* side population cells, *eMSCs* endometrial mesenchymal stem cells

CD146^+^ cells derived from human endometrium can form colony-forming units [18] and differentiate into adipocytes, osteoblasts, neural progenitors, and glial-like cells [19, 20] (Table 1). With the help of the collagen–matrigel scaffold on the top of the myometrial smooth muscle cells, human endometrial CD146^+^ cells may generate endometrial gland-like structures in vitro [21] and express all recognized markers of MSCs, including CD10, CD13, CD44, CD73, CD90, and CD105 [20] (Table 1). Cysteine-rich angiogenesis inducer 61 (CYR61), also called CCN family member 1, is highly expressed in endothelial cells and smooth muscle cells [22] and may play an important role in angiogenesis and tissue repair [23, 24]. Compared with CD146^+^CYR61^−^, CD146^+^CYR61^+^ cells can stimulate angiogenesis. The rat endometrium transplanted with CD146^+^CYR61^+^ cells appear with higher blood vessel density than that transplanted with CD146^+^ or CD146^+^CYR61^−^ cells. In addition, endometrial injury rats transplanted with CD146^+^CYR61^+^ cells appear with higher pregnancy rate than control group [20].

### SUSD2^+^ eMSCs

SUSD2, a novel marker of eMSCs, is proved particularly effective in the selection of eMSCs [12]. SUSD2^+^ cells reside predominantly in a perivascular location in both basal and functional layers of endometrium (Fig. 1). SUSD2^+^ cells can differentiate into adipocytes, osteocytes, chondrocytes, myocytes, endothelial cells in vitro and produce endometrial stromal-like tissues in vivo (Table 1). Freshly isolated SUSD2^+^ cells express MSC markers including CD29, CD44, CD73, CD90, CD105, CD117, CD140b, CD146, and STRO-1 (Table 1). SUSD2^+^ cells also express nucleoside triphosphate diphosphohydrolase 2 (NTPDase2), a membrane-expressed enzyme existing in mesenchymal-derived cells, such as pericytes in different tissues and stem cells in adult neurogenic regions [25, 26]. The expression level and localization of NTPDase2 remain unchanged throughout the menstrual cycle, indicating that the enzyme can be used as a cell marker to improve the separation of eMSCs for regenerative medicine treatment [27].

SUSD2^+^ eMSC seems to be affected by pregnancy and obesity, but not by aging. In the undifferentiated state, SUSD2^+^-derived cells produce lower levels of various chemokines and inflammatory regulators than SUSD2^−^ cells. However, this is switched after decidualization because these SUSD2^+^ cells are turned into the main source to produce chemokines and cytokines including chemokine (C–C motif) ligand 7, and the leukemia inhibitory factor [28]. SUSD2^+^ cells originated from myometrium and uterine fibroids are featured as MSCs and can also be induced into decidua [29]. Perivascular SUSD2^+^ cells isolated from postmenopausal endometrium also display the characteristics of MSCs, regardless whether the patients receive estrogen pretreatment for the regeneration of endometrium [30]. However, adipocytes may adversely affect endometrial stem cells. Compared with that in women with normal body mass index (BMI), the proportion and cloning efficiency of SUSD2^+^ cells in the endometrium of obese women are significantly reduced [31].

### Signaling pathways involved in SUSD2^+^ eMSCs

**Fig. 2:**
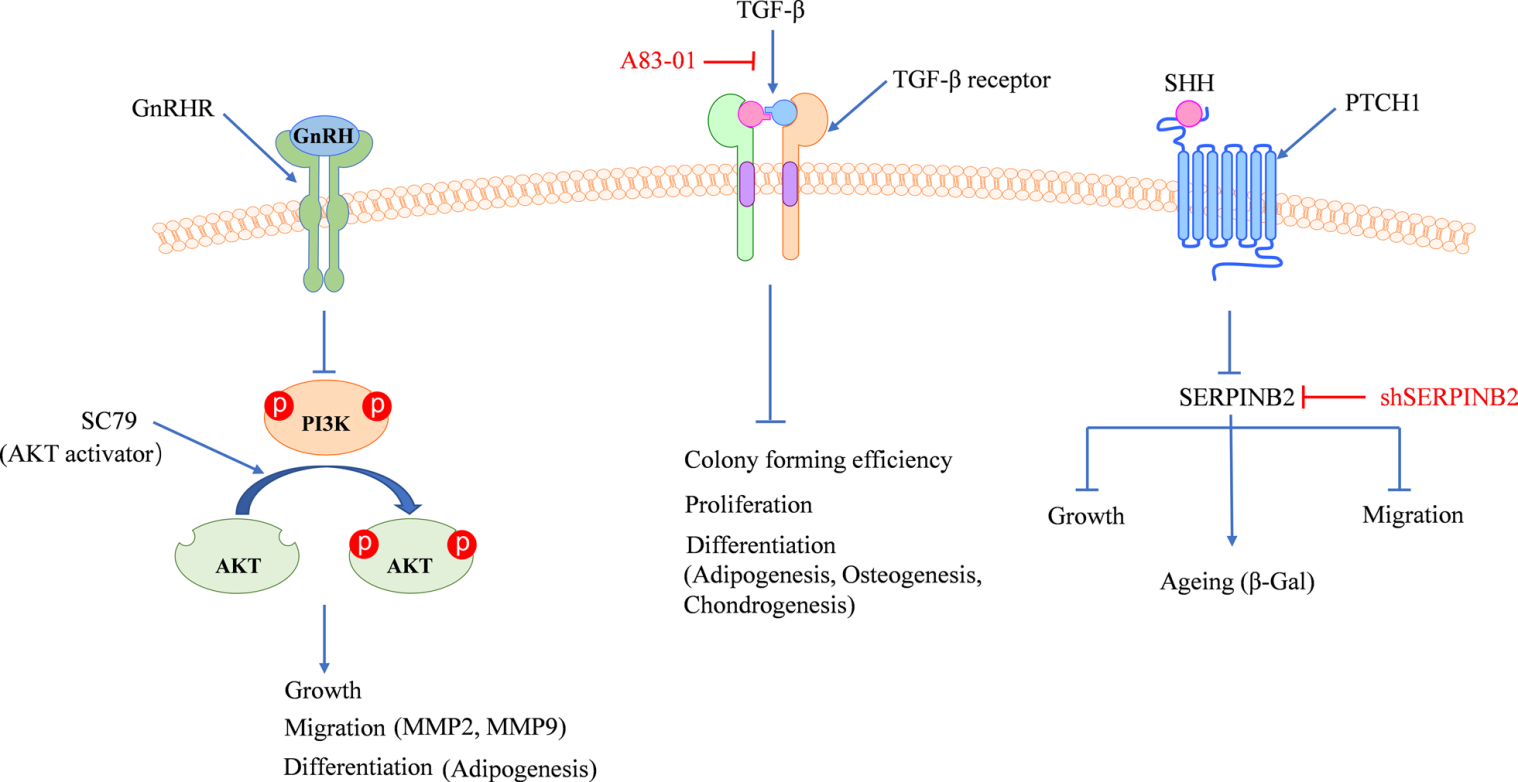
GnRH, TGF-β, and SHH affect the multiple functions of eMSCs, such as proliferation, differentiation, aging, and migration. GnRH inhibits the multiple beneficial functions of eMSCs, such as proliferation, differentiation, and migration, through the PI3K/AKT signaling. The activation of Akt signaling attenuates the GnRH-induced adverse effects on multiple stem cell functions. TGF-β inhibits the proliferation, differentiation, and colony-forming efficiency of SUSD2^+^ eMSCs. A83-01, TGF-β receptor inhibitor, can maintain the clonogenicity of SUSD2^+^ eMSCs, promote proliferation, prevent cell apoptosis, and maintain eMSC function. Exogenous SHH therapy could significantly alleviate various aging-related declines in multiple eMSC functions through the inhibition of SERPINB2 expression

In recent years, scientists have gradually paid the attention to the clinical application of endometrial stem cells. The in vitro expansion and stemness maintenance of eMSCs are a major challenge for the current clinical treatment. Studies have found that A83-01, a TGF-β receptor inhibitor, can maintain SUSD2+ eMSCs proliferation, clonogenicity, and function  through the inhibition of TGF-βR signaling [32, 33] (Fig. 2). The expression of genes associated with anti-inflammatory response, angiogenesis, cell migration and proliferation can be promoted by A83-01 in SUSD2^+^ eMSCs [34].

Long-term GnRH exposure of eMSCs may be responsible for the relatively low rate of in vitro fertilization (IVF) positive pregnancy outcomes. Unlike terminally differentiated fibroblasts, SUSD2^+^ eMSCs express abundant GnRH receptors. GnRH inhibits the multiple beneficial functions of eMSCs, such as proliferation, differentiation and migration, through the PI3K/Akt signaling pathway [35] (Fig. 2).

The Sonic hedgehog (SHH) signaling typically functions in morphogenesis during the embryonic development [36]. In addition, the decreased SHH signal integrity of local eMSCs may be a potential factor for the decreased regeneration of ageing endometrium. The activity of SHH is decreased significantly with ageing, but the exogenous SHH therapy may significantly alleviate the various ageing-associated declines. SERPINB2 is a major regulator for the SHH signal transduction during senescence, whereas the senescence of stem cells may enhance the expression of SERPINB2, which in turn mediates the role of SHH to attenuate the senescence-induced dysfunction of eMSCs [37] (Fig. 2).

### SUSD2^+^ eMSCs in immunity and tissue engineering

**Fig. 3:**
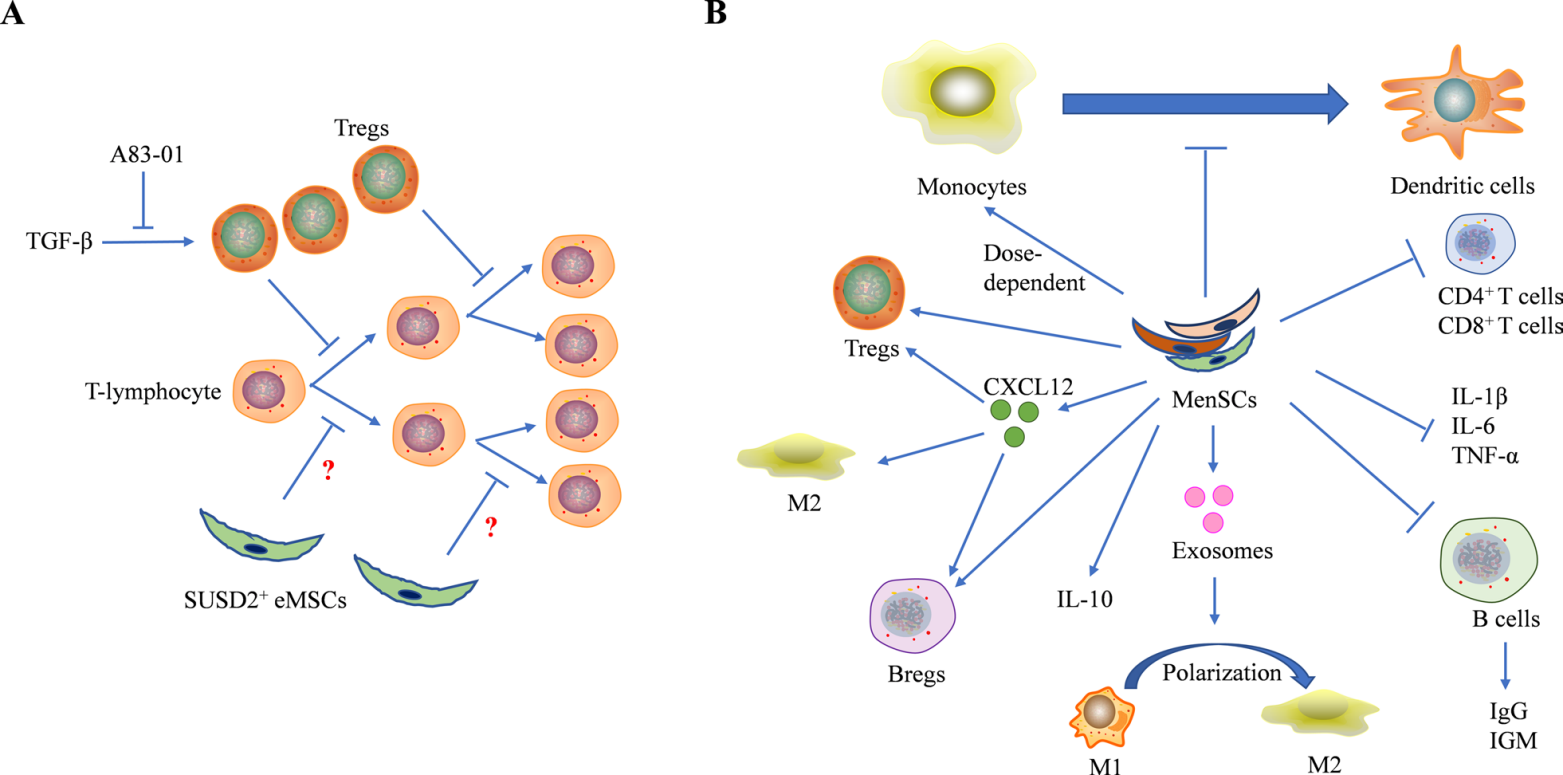
Roles of SUSD2^+^ eMSCs and MenSCs in immunity. **A** TGF-β promotes the differentiation of Tregs that inhibit T-lymphocyte proliferation. A83-01 increases the T-lymphocyte proliferation through the inhibition of the TGF-β signaling-dependent Treg differentiation, but SUSD2^+^ eMSCs continue to inhibit the lymphocyte proliferation via an uncertain mechanism independent of the TGF-β signaling from that of MSC from other tissues. **B** MenSCs inhibit the phenotypic differentiation of human peripheral blood monocytes into immature and mature DCs. MenSCs can also affect the proliferation of monocytes in a dose-dependent manner. In vivo studies, after the intravenous injection of MenSCs, the proportion of CD4^+^ and CD8^+^ T cells in spleen was significantly down-regulated and the percentage of CD4^+^CD25^+^Foxp3^+^ regulatory T cells (Treg) and Breg (CD19^+^IL‐10^+^) in spleen was significantly up-regulated. The serum levels of IL-1β, IL-6, and TNF-α in mice receiving MenSCs transplantation are lower, but the expression level of IL-10 is higher. CXCL12 secreted by MenSCs also increases the percentage of Treg, Breg, and M2 cells. MenSC-derived exosomes can resolve inflammation through the induction of the M1-M2 macrophages polarization. MenSCs treatment may inhibit the proliferation of B cells to reduce the production of IgM and IgG antibodies

Mesenchymal stem cells (MSCs) from other tissues, such as bone marrow, umbilical cord, and adipose tissues, inhibit the proliferation of T cells, B cells, natural killer cells (NK), and dendritic cells (DCs) to induce cell cycle arrest through the mechanisms associated with IL-10, prostaglandin E2, TGF-β1, and regulatory T cells (Tregs) [38]. Although SUSD2^+^ eMSCs inhibit the mitogen-induced lymphocyte proliferation in a dose-dependent manner, blocking of the mouse IL-10 receptors or the prostaglandin production dose not inhibit lymphocyte proliferation. Despite the reduction of Tregs, endometrial SUSD2^+^ cells continue to inhibit lymphocyte proliferation in the presence of TGF-β receptor inhibitors [39]. Therefore, the inhibition of the mitogen-induced lymphocyte proliferation by SUSD2^+^ cells occurs through an uncertain mechanism different from that of MSCs from other tissues (Fig. 3A). Moreover, the systemic administration of endometrial SUSD2^+^ cells dose not inhibit the swelling of the T cell-mediated skin inflammation. Although endometrial SUSD2^+^ cells can alter the immune response, their immunoregulatory pool may not be sufficient to suppress the certain T cell-mediated inflammatory events [39].

Animal studies demonstrate that SUSD2^+^ eMSCs can also modify immune responses to the implanted mesh [39]. Seeding of eMCSs in scaffolds can promote the formation and reconstruction of neo-tissues [40, 41]. The eMSCs alter the growth of collagen and organization around the mesh filaments of the scaffold to affect the physiologically relevant tensile properties of the scaffold-tissue complex. The stiffness of scaffolds seeded with eMSCs on initial stretching can be significantly alleviated. In addition, the scaffold is an appropriate platform for eMSCs delivery, proliferation, and differentiation, with the better biocompatibility and the capacity to regenerate neo-tissues, which may be a promising application in the clinical mesh repair of pelvic organ prolapse (POP) to reduce the excessive scar tissue formation induced by foreign body reactions and to relieve the in vivo poor mechanical compliance.

## Menstrual stem cells

Menstrual stem cells (MenSCs) were first identified from menstrual blood in 2007, which can effectively propagate for over 68 population doublings with normal karyotype [42]. MenSCs express markers CD29, CD9, CD13, CD44, CD41a, CD73, CD59, CD90, and CD105 but not CD19, CD34, CD45, CD117, CD130, or HLA-DR [42, 43] (Table 1). MenSCs partially (over 50%) express the pluripotency marker SSEA-4, but not Oct-4. MenSCs can differentiate into adipocytic [44], osteogenic [45], cardiomyocytic [46], and neurocytic lineages [47], as well as respiratory epithelial, endothelial, myocytic, hepatic [48], germ-like [49, 50], and pancreatic cells [42, 51] (Table 1). Replacement of fetal bovine serum with human platelet derivatives can promote the differentiation of MenSCs into osteoblasts [52]. The mitotically inactivated MenSCs are ideal feeder cells for the human embryonic stem cell lines C612 and C910 [43].

### MenSCs in regenerative medicine and tissue engineering

MenSCs population is one of the clinically accessible sources of stem cells with great potential in regenerative medicine. MenSCs are abundant in sources with excellent proliferation and autotransplantation capabilities and can be collected regularly and noninvasively. In addition, MenSCs have a higher proliferation ability than that of BMSCs [53]. Most importantly, any significant side effects including acute, subchronic, or chronic poisoning, infection, tumorigenesis, or endometriosis has not been reported either in preclinical studies or in clinical studies during the treatments of various diseases with MenSCs over the past yeas [54–56] (Table 2). Existing studies have found that MenSCs therapy may be an attractive alternative approach for intrauterine adhesion (IUA) [57], premature ovarian failure (POF) [58, 59], liver failure [60–62], experimental stroke [63], pulmonary fibrosis [64, 65], cardiac diseases [66, 67], myocardial infarction [46, 68], Alzheimer’s disease [69], acute lung injury [70], acute respiratory distress syndrome [71], renal ischemia reperfusion injury [72], sciatic nerve injury [73], chronic nonhealing wounds [74], and type 1 diabetes [75] (Table 2).

**Table 2:** Some of the disorders could be (or already are) treated by MenSCs

Disorder	Subjects	References
IUA	Human	[57]
	Rat model	[151]
Endometrial injury	Mice model	[152]
Premature ovarian failure	Rat model	[58]
	Mice model	[59, 78]
Liver failure	Mice model	[60–62]
	Pig model	[153]
Liver fibrosis	Mice model	[154]
Experimental stroke	In vitro stroke model of oxygen glucose deprivation	[63]
Pulmonary fibrosis	Mice model	[64, 65]
ARDS	Patients with H7N9-induced ARDS	[71]
Myocardial infarction	Rat model	[46, 68]
Cardiac allograft	Mice model	[67, 90]
Alzheimer’s disease	Mice model	[69]
Acute lung injury	Mice model	[70]
Renal ischemia reperfusion injury	Mice model	[72]
Type 1 diabetes	Mice model	[75]
Chronic nonhealing wounds	Diabetic mice model	[74]
Sciatic nerve injury	Rat model	[73]

*MenSCs* menstrual stem cells, *IUA* intrauterine adhesion, *ARDS* acute respiratory distress syndrome

Studies reported that MenSCs may be used for patients with severe IUA. MenSCs co-cultured with endometrial stromal cells (ESCs) promote the proliferation and wound repair of ESCs, down-regulate the expression of αSMA and collagen I in ESCs, and reverse the fibrotic gene expression in ESCs induced by TGF-β through the Hippo/TAZ signaling pathway [76]. Intrauterine transplantation of MenSCs in the IUA rat model demonstrate that the endometrial pathology and uterine fertility of the rat are significantly improved [77]. Human autologous MenSCs transplantation may significantly promote the endometrial morphology regeneration and functional recovery in patients with severe IUA, which thereby helps some patients achieve a positive pregnancy [57].

MenSCs with properties of high survival rate in vivo and easy access make them very useful for stem cell transplantation in POF therapy. By two-dimensional culture and 3D scaffold culture system, MenSCs can differentiate into germ-like cells in vitro [49, 50]. MenSCs transplantation increases the body weight of POF mice, improves the estrus cycle, and restores the fertility of POF mice [78]. The transplanted MenSCs can be detected in the ovarian stroma and survive in the ovaries of POF mice for at least 14 days [59,78], and can be differentiated into granulosa cells and traced to two months in the ovaries of POF rats [58]. The ovaries receiving MenSCs transplantation express the higher levels of ovarian reserve markers (AMH, inhibin α/β, and follicle-stimulating hormone receptor) and increase the ovarian weight, the plasma E2 level, and the normal follicle counts [59].

The application of MenSCs in tissue engineering is also promising. A wide variety of 3D scaffolds has been applied to induce differentiation and co-culture of MenSCs. On the nanofiber scaffolds with the specific growth and differentiation factors, MenSCs may be differentiated into chondrocytes to anchor firmly on the highly porous scaffold, and to penetrate and spread on the scaffold. The scaffold contains an extensive cartilage-like extracellular matrix whose glycosaminoglycan content is about 50% higher than that of the 2D culture system through which MenSCs differentiated [79]. On the 3D wet-electrospun poly (lactic acid)/multi-wall carbon nanotube scaffold, MenSCs can be differentiated into germ-like cells [50]. Based on the bilayer amniotic membrane/nano-fibrous fibroin scaffold, MenSCs can be differentiated into keratinocyte like cells in the presence of keratinocytes derived from human foreskin [80]. In the 3D co-culture system of mouse preantral follicles and human MenSCs, the follicular growth indices are significantly increased, including survival rate, diameter and antrum formation as well as the rate of in vitro maturation rate [81].

### Interaction of MenSCs with immune cells

MenSCs interact with a variety of immune cells and participate in the regulation of cellular immunity and humoral immunity (Fig. 3B). Menstrual blood can be used not only as a source of MenSCs, but also as a source of DCs. Monocytes in menstrual blood can be induced into DCs by a two-step protocol [82]. DCs, the professional antigen-presenting cells, may form an indispensable interface between the innate sensing of pathogens and the activation of adaptive immunity, which thereby enables DCs to be used as a novel and promising immunetherapeutic approach for cancer, persistent infection and autoimmune diseases treatment [83–85]. Similar to SUSD2^+^ eMSCs, MenSCs inhibit the optimal phenotypic differentiation of human peripheral blood monocytes (PBMCs) into immature and mature DCs, in which IL-6 and IL-10 may play an important role [86]. Moreover, MenSCs may also affect the proliferation of monocytes in a dose-dependent manner [87]. The immunosuppressive effects of MenSCs on PBMCs, CD4^+^IFN-γ^+^, and CD8^+^IFN-γ^+^ cells are weaker than those of BMDSCs, but MenSCs appear with a higher capacity to migrate into the intestine and liver [88].

In vivo studies showed that MenSCs may protect mice liver from acute injury through the anti-inflammatory and immunomodulatory effects. In the mice model with acute injury liver, the proportion of CD4^+^ and CD8^+^ T cells in spleen was significantly down-regulated after intravenous injection of MenSCs, while the percentage of CD4^+^CD25^+^Foxp3^+^Tregs in spleen was significantly up-regulated. Additionally, the splenic DCs in MenSCs-treated mice displayed a significant decrease of the MHC-II expression. The serum and liver levels of IL-1β, IL-6, and TNF-α in mice receiving MenSCs transplantation are lower, but the expression level of IL-10 is higher [60]. In the colitis mice model, the treatment with MenSCs mainly regulated the response of B-lymphocytes, whereas the intravenous injection of MenSCs decreased the percentage of immature plasma cells in spleen and IgG deposition in colon but increased the secretion of IL-10 and the production of Bregs (CD19^+^IL-10^+^) [89]. On wound-healing process, MenSCs-derived exosomes can attenuate inflammation through the induction of the M1-M2 macrophage polarization [74].

The therapeutic function of MenSCs used to alleviate the antibody-mediated allograft rejection can be partly attributed to the cellular immunity regulation [67] and the humoral immunity suppression [90]. The MenSC-mediated therapy can prolong the survival of the mice receiving cardiac allotransplantation due to the decrease of IgM and IgG deposition and the circulation of the anti-donor antibodies secreted by CD19^+^ B cells. In addition, by ex vivo stimulation, because the proliferation of B cells from the MenSC-treated heart transplant recipients is impaired, and the production of IgM and IgG antibodies is reduced [90]. Stromal-cell-derived factor‐1 (SDF‐1), also known as CXCL12, can be secreted in a substantial amount by MenSCs. The MenSC-mediated therapy can induce immunosuppression and donor-specific allograft tolerance in which the SDF-1 secreted by MenSCs plays important roles. Based on MenSCs therapy, SDF-1 can reduce the antibody-mediated rejection and acute cellular rejection to increase the percentages of Tol-DC (CD11c^+^MHC class II^+^), Treg (CD4^+^CD25^+^Foxp3^+^), Breg (CD19^+^IL‐10^+^), and M2 (CD68^+^CD206^+^) cells, and to reduce the percentage of total macrophages [67]. As easily accessible and expandable stem cells, MenSCs are worthy of the researchers’ attention for their functions in the regulation of the immune system-related cells and humoral immunity.

## Side population cells

Side population cells (SPs) are considered a universal marker for adult stem cells in mammalian species. This phenotype results from the high expression of plasma membrane transporters (such as ABCG2), which transports the DNA-binding dye Hoechst 33,342 out of the cell [91]. SPs were first isolated from normal human endometrial cells by Kato et al. in 2007 and can be differentiated into gland- and stromal-like cells [92]. Human endometrium contains approximately 1–7% SPs in freshly isolated human endometrial at various stages, including proliferative phase [93], secretory phase and decidual of early pregnancy [94, 95]. Most SPs in the endometrium are resting cells in vivo, but during the proliferative phase, a small number of SPs become active to be differentiated into endometrial cells [93, 94]. SPs are located at the vascular endothelium cells lining blood vessels in both the functionalis and the basalis of the endometrium [94] (Fig. 1).

Specific markers have been identified for SPs (Table 1). Endometrial SPs are composed of heterogeneous populations, with endothelial cell markers (CD31), hematopoietic cell markers (CD34 and CD45), the epithelial cell marker EMA and mesenchymal stem cell markers (CD90, CD105, and CD146) [94, 96, 97]. The enrichment of endothelial and CD146^+^CD140b^+^ eMSCs suggests that the endometrial SPs play a role in angiogenesis during the endometrial regeneration [98]. However, SPs in human decidua of early pregnancy are negative for CD13, CD34, and CD45, but about 95% of SP cells in human decidua are CD31^−^CD146^−^ [99] (Table 1). No difference in the percentage of SUSD2^+^ cells exist between the endometrial SP and non-SP components, but CD140b^+^ CD146^+^ cells are much more abundant in endometrial SPs than in non-SP components [100]. With the greater colony-forming efficiency than non-side population cells [94], SPs can be differentiated into various types of endometrial cells, such as stroma, glandular epithelium, and endothelium cells [93], adipocytes and osteoblasts [96, 101]. SPs also rebuild the well-organized endometrial tissues and glandular structures in vivo [93, 96, 97, 100, 102].

Although the endometrial SPs are featured with the excellent self-renewal and differentiation abilities, the dynamic labeling is technically difficult to be performed, the co-labeling with other markers is unreliable, the Hoechst dye is toxic to cells, and flow cytometry sorting damages cells [14, 103]. Therefore, the heterogeneity of the SPs and their isolation method hinder their clinical applications.

## Endometrial epithelial stem/progenitor cells

Endometrial epithelial progenitor cells were first isolated by Gargett et al. [15]. Individual colonies in the differentiation induction medium are characterized as adult stem cells by analysis of the self-renewal, differentiation, and high proliferative potential of single epithelial. The stage-specific embryonic antigen-1 (SSEA-1), as a marker of human endometrial basal glandular epithelial cells, is used to distinguish the epithelium of basalis from functionalis [104, 105] (Fig. 1). SSEA-1^+^ endometrial epithelial cells displaying some characteristics of the basalis epithelium and the higher telomerase activity may produce a higher number of endometrial gland-like spheroids than SSEA-1^−^ endometrial epithelial cells in 3D culture system.

Recently, through in vivo lineage tracking, researchers found that the endometrial epithelium maintains the continuous self-renew during the development, normal growth, and regeneration of the whole life, and demonstrated that a multipotent endometrial epithelial stem cells with naturally occurring somatic mitochondrial DNA mutations (CCO gene) can regenerate the entire complement of glandular lineages [106, 107]. Axin2, a key negative regulator of the Wnt signaling pathway is expressed in the stem cells of various organs [108], and is also identified as a marker of long-lived bipotent epithelial progenitors that reside in endometrial glands [107]. Cytoplasmic Axin2 is also expressed in the functionalis of proliferative and secretory endometrial glandular epithelia from premenopausal women. In contrast, the nuclear Axin2 expression is observed in the proliferative and secretory basalis of premenopausal and postmenopausal endometrial epithelia [105]. Axin2-expressing glandular cells express the known stem cell markers, such as Lgr5, Trop2 and Sox9 to fuel endometrial epithelial growth and regeneration in vivo. In addition, Axin2^+^ cells can form fully functional endometrial organoids in vitro [107]. The above findings seem to provide evidence for the involvement of the mesenchymal-to-epithelial transition (MET) in the maintenance and regeneration of the uterine epithelium [109]. However, a recent cell fate tracing study found that the conclusive evidence for the conversion of mesenchymal cells to epithelial cells in adult uterine is lacking. The study of the embryonal cell lineage tracing with reporters driven by mesenchymal cell marker genes of the female reproductive tract (AMHR2, CSPG4, and PDGFRβ) showed that these reporters are also expressed in some oviductal and uterine epithelial cells at birth [110].

The endometrial epithelial stem cell population of mouse residing in the intersection zone between luminal and glandular epithelial compartments is also identified by in vivo lineage tracking in which the tissue distribution allow the bipotent endometrial epithelial stem cells to be differentiated bidirectionally into luminal epithelial cells and glandular epithelial cells and to maintain the homeostasis and regeneration of the mouse endometrial epithelium under physiological conditions [111]. However, no labeled epithelial cells were found in any fallopian tubes or uterine epithelium after the mesenchymal cell labeling is induced in adult mice, indicating that no definitive evidence of MET happens in the fallopian tubes and uterine epithelium in murine [110]. Very small embryonic-like stem cells (VSELs) are recently identified in mouse uterine [112], but they are still controversial [113] because without the sufficient functional analysis to prove their pluripotency until now [4].

## Participation of endometrial stem/progenitor cells in the origin and development of endometriosis

Endometriosis is characterized by the development of endometrial tissues outside the uterus to cause pain and infertility. Due to the lack of effective biomarkers, endometriosis is usually not diagnosed until the first onset of the disease a few years later. So far, most of the existing treatments are non-therapeutic [8]. Until the beginning of the twenty-first century, some scholars suspected that endometriosis may be a stem cell-related disease, because less differentiated endometrial cells in RM may be the cellular source of primary endometriotic lesions [8, 114, 115]. Endometrial stem/progenitor cells with the altered molecular properties reflux into the pelvic cavity via RM, where they adhere and form ectopic lesions. The prevalence of shed basalis fragments in the menstrual blood of women with endometriosis is significantly higher than that in the healthy control menstrual blood [8]. The endometrium of endometriotic lesions displays a cyclical pattern similar to the basalis and presents the same cyclical pattern of ER and PR expression as the deep basalis. The expression of adult stem cell markers Musashi-1 [116], OCT4, SOX15, SOX2 [117, 118], C-kit [119], Notch and Numb [120], and the corneal epithelial progenitor cell marker importin13 [121] is significantly higher in endometriotic lesions than in normal endometrium. The peripheral lymphocytes from endometriosis patients are detected with longer telomeres than those from healthy controls [122]. Moreover, the expression of SSEA-1 in ectopic epithelial cells is similar to that in eutopic basalis epithelium [104, 123]. These data support the concept of a stem cell origin of endometriosis that the presence of the abnormally detached basalis endometrium fragments in the RM is considered as the main cause of endometriosis (Fig. 1).

## Peritoneal microenvironment interacts with ectopic cells in patients with endometriosis

Endometriosis alters the peritoneal microenvironment of women, in which the immune response, angiogenesis, cell proliferation, cell adhesion, and apoptosis are uniquely regulated in peritoneal fluid (PF). A specific protein expression pattern is present in PF with deep infiltrating endometriosis (DIE) compared in PF with non-DIE [124]. The detached endometrial fragments flow into the pelvic cavity, where they directly interact with cytokines in PF [125] to secrete chemokines [126] and to form a feedforward loop [127], which eventually induces the infiltration of immune cells and BMDSCs [128]. Seventy-four cytokines are increased and 4 cytokines are decreased in PF from endometriosis patients compared with those in healthy control group [125]. Among these cytokines, activin A is significantly increased in PF from endometriosis group, whereas ALK4 (activin A-specific receptor) is increased in ectopic endometrial-derived SUSD2^+^ eMSCs [129]. In addition, the levels of Activin A secreted by glandular cells and stromal cells are significantly higher in the eutopic endometrium of endometriosis patients than in the eutopic endometrium of healthy controls [130]. The expression of the connective tissue growth factor (CTGF) in SUSD2^+^ eMSCs may be promoted by Activin A through the binding of Smad2/3 to the CTGF promoter to induce the myofibroblast differentiation of SUSD2^+^ eMSCs. Endometriotic lesions may be enhanced by Activin A through the increased IL-6, IL-8, and TNF-α in the ascites of endometriosis mice models [131, 132]. Inhibition of the activin A pathway prevents the myofibroblast differentiation of SUSD2^+^ eMSCs and improves fibrosis in endometriosis mice [129]. Endometriotic cells interact with the abnormal peritoneal microenvironment of patients with endometriosis. The ectopic cells secrete inflammatory factors that may remodel the peritoneal microenvironment, and in turn, various cytokines in PF exert their function on the endometriotic cells.

## Abnormal expression profiles of endometrial stem cells from endometriosis patients

Ectopic eMSCs from endometriosis patients display stronger abilities of proliferation, migration, and angiogenesis than eutopic eMSCs from the same individual or from healthy controls [133]. The expression profiles of adenomyosis-derived mesenchymal stem cells (AMSCs) are different from those of eMSCs and BMSCs. Compared with eMSCs, the expression of cyclooxygenase-2 (COX-2) in AMSCs is significantly increased, and inhibition of COX-2 blocks the migration and invasion of AMSCs and induces their apoptosis [134].

CD73^+^CD90^+^CD105^+^ endometrial stem cells (SCs^+^) from normal, ectopic and eutopic endometrium display a significantly higher level of SUSD2^+^ with cloning efficiency and sphere formation capacity than SCs^−^. Compared with in eutopic endometrium SC^+^ samples, the expression of PTEN, ARID1A, and TNFα from paired-ectopic samples is significantly down-regulated. Analysis of the hierarchical and multivariate clustering from both SC^+^ and tissue cohorts revealed the abnormal expression of stemness-related and cancer-related genes such as KIT, HIF2α, and E-Cadherin in 4 of 30 ectopic samples. C-kit is expressed higher in the endometrial glandular cells of the women with endometriosis than in the endometrial glandular cells of the women without endometriosis [119]. Therefore, it is speculated that the changes in stemness-associated genes may be linked to the development of endometriosis [135].

MenSCs from women with and without endometriosis display different phenotypic and functional characteristics [136]. MenSCs from the endometriosis (E-MenSCs) women appear with the higher expression of CD9, CD10, and CD29 and the higher proliferation and invasion potentials than MenSCs from the non-endometriosis (NE-MenSCs) women. The expression of the indoleamine 2,3-dioxygenase-1 (IDO1) and COX-2 in E-MenSCs is higher than in NE-MenSCs. In addition, the supernatants of E-MenSCs contain the higher levels of IFN-γ, IL-10, and the monocyte chemoattractant protein 1 than those of NE-MenSCs. These findings indicate that MenSCs may play an alternative role in the pathogenesis of endometriosis, which further supports the stem cell theory of endometriosis with RM.

## Stem/progenitor cells or stem-like cells of extrauterine origin promote endometriosis

A study reported that a few of stromal cells and epithelial cells from doner mouse endometrial tissues were traced in the ectopic implant lesions of the recipient mice after 10 weeks of transplantation, indicating that the cells from the extrauterine origin may also promote the development of ectopic endometrium [137].

BMDSCs participate in the pathogenesis of endometriosis to promote the development of the disease [138] (Fig. 1). BMDSCs implanted into ectopic endometrial and endometriotic lesions display the properties of stromal and epithelial cells [137, 139], while the cytokines secreted by the implanted BMDSCs promote the proliferation of ectopic endometrial cells [138]. In turn, the endometriotic cells also stimulate the BMDSCs differentiation and increase the expression of PD-1 in T cells possibly through the paracrine signaling [140]. The ectopic endometrium competes with the eutopic endometrium for the limited supply of BMDSCs in blood circulation and the depletion of normal BMDSCs flux to the uterus. In addition, stem cells migrate from the endometriotic lesions to the uterus, to induce the dysfunction of the eutopic endometrium [141]. 17β-Estradiol can promote the chemotaxis and migration of BMDSCs by up-regulating the secretion of chemokine SDF-1α [142]. In a mouse endometriosis model, bazedoxifene [139], an estrogen receptor modulator, administered with the conjugated estrogens and letrozole [143] (aromatase inhibitor) not only alleviated the lesions of endometriosis, but also dramatically reduced the recruitment of BMDSCs to the lesions and restore the stem cell engraftment of the uterine endometrium.

Endometrial stromal cells express the chemokine CXCL12, while BMDSCs express CXCR4, the receptor of CXCL12 [144]. In human and mice models of endometriosis, higher levels of CXCL12 and CXCR4 were detected in ectopic lesions and serum than those in healthy controls [145]. The fluctuation of CXCL12 concentration produces a chemical gradient that guides the migration of stem cells [146]. The chemoattraction of mouse BMDSCs to CXCL12 in the conditioned medium (CM) of endometriotic cells is higher than that in the CM of eutopic endometrium [145]. Activation of the CXCL12/CXCR4 signaling axis promotes the ectopic lesions to outcompete eutopic endometrium to recruit the limited supply of circulating BMDSCs. Targeting CXCR4 by using the small molecule receptor antagonist AMD3100 reduces the recruitment of BMDSCs into the endometriosis and the size of the endometriosis lesions [147]. Antagonist treatment also reduces the production of pro-inflammatory cytokines and angiogenesis in the lesions of endometriosis [147].

Circulating endometrial cells (CECs) were identified in the peripheral blood of all the acknowledged endometriosis stages: minimal, mild, moderate, and severe (Fig. 1). The CECs captured during the menstrual cycle phases display stem cell-like characteristics [148]. CECs are also found in the patients with pelvic endometriosis and spontaneous pneumothorax, with the properties of epithelial, stroma-like, glandular [149], or stem cell-like cells. A reporter found that DsRed^+^ cells can be found in blood of DsRed^−^ mice with endometriosis receiving the peritoneal cavity transplantation of DsRed+ mice endometrial tissues. Almost all of CECs originated from endometriosis rather than uterus express CXCR4 and MSCs biomarkers, but not hematopoietic stem cell markers, and contribute to both endometriosis and angiogenesis. Cells originated from endometriosis lesions may migrate and implant in lung tissues and display the abilities of differentiation into adipogenic, osteogenic, and chondrogenic lineages in vitro, indicating a retained multipotency.

Overall, endometrial stem/progenitor cells in menstruation blood (MenSCs) are the most clinically accessible sources of stem cells with a great potential in the regenerative medicine and tissue engineering. The advantages of MenSCs are that they can be collected regularly and noninvasively. MenSCs are also promising candidates in the stem cell therapy for inflammation and immune-related diseases, and may play an immunosuppressive role in the regulation of the cell-mediated immunity and humoral immunity. The bone marrow-derived and endogenous stem/progenitor cells participate in the origin and development of endometriosis. Endogenous stem/progenitor cells with the altered molecular properties from the shedding endometrium fragments may reflux into the pelvic cavity via RM, which may be considered as the main inducer of endometriosis. The ectopic lesions compete with the eutopic endometrium for the limited BMDSCs in blood circulation to induce the establishment of the deep invasive endometriosis. In addition, stem-like cells in ectopic lesions may also enter the peripheral blood circulation and cause distant invasion. The study of the molecular mechanisms of stem/progenitor cells or stem-like cells in endometriosis may provide some promising targets for molecular therapy of the associated reproductive and cancerous diseases.

## Data Availability

Not applicable.

## Abbreviations

eMSCs: endometrial mesenchymal stem cells
SPs: side population cells
MenSCs: menstrual stem cells
BMDSCs: bone marrow mesenchymal stem cells
RM: retrograde menstruation
CFUs: colony-forming units
LRCs: label-retaining cells
ABCG2: ATP-binding cassette transporter G2
Erα: estrogen receptor alpha
PR: progesterone receptor
SSEA-1: stage-specific embryonic antigen-1
MSC: mesenchymal stem cell
CXCL1: C-X-C motif ligand 1
CYR61: cysteine-rich angiogenesis inducer 61
NTPDase2: nucleoside triphosphate diphosphohydrolase 2
BMI: body mass index
IVF: in vitro fertilization
SHH: Sonic hedgehog
POP: pelvic organ prolapse
PCL: poly ε-caprolactone
IUA: intrauterine adhesion
ESCs: endometrial stromal cells
POF: premature ovarian failure
DCs: dendritic cells
PBMCs: peripheral blood mononuclear cells
SDF‐1: stromal cell‐derived factor‐1
DIE: deep infiltrating endometriosis
PF: peritoneal fluid
CTGF: connective tissue growth factor
AMSCs: adenomyosis-derived mesenchymal stem cells
COX-2: Cyclooxygenase-2
SC^+^: CD73^+^CD90^+^CD105^+^ multipotent stem cell
IDO1: indoleamine 2,3-dioxygenase-1
CECs: Circulating endometrial cells
MET: mesenchymal-to-epithelial transition
